# Patient Preferences for Longer or More Frequent In-Center Hemodialysis Regimens: A Multicenter Discrete Choice Study

**DOI:** 10.1053/j.ajkd.2021.09.012

**Published:** 2022-06

**Authors:** James Fotheringham, Enric Vilar, Tarun Bansal, Paul Laboi, Andrew Davenport, Louese Dunn, Arne Risa Hole

**Affiliations:** 1 School of Health and Related Research, University of Sheffield, Sheffield, United Kingdom; 2 Department of Economics, University of Sheffield, Sheffield, United Kingdom; 3 Sheffield Kidney Institute, Northern General Hospital, Sheffield, United Kingdom; 4 University of Hertfordshire, Hatfield, United Kingdom; 5 Bradford Teaching Hospitals NHS Trust, Bradford, United Kingdom; 6 York Teaching Hospital NHS Foundation Trust, York, United Kingdom; 7 UCL Department of Nephrology, Royal Free Hospital, University College London, London, United Kingdom

**Keywords:** Dialysis regimen, discrete choice experiment (DCE), end-stage renal disease (ESRD), frequent dialysis, goals of care, health economics, intensive dialysis, longer dialysis, patient-centered care, patient preference, quality of life (QoL), treatment burden, shared decision making

## Abstract

**Rationale & Objective:**

Longer and more frequent hemodialysis sessions are associated with both benefits and harms. However, their relative importance to patients and how they influence acceptability for patients have not been quantified.

**Study Design:**

Discrete-choice experiment in which a scenario followed by 12 treatment choice sets were presented to patients in conjunction with varying information about the clinical impact of the treatments offered.

**Setting & Participants:**

Patients with kidney failure treated with maintenance dialysis for ≥1 year in 5 UK kidney centers.

**Predictors:**

Length and frequency of hemodialysis sessions and their prior reported associations with survival, quality of life, need for fluid restriction, hospitalization, and vascular access complications.

**Outcome:**

Selection of longer (4.5 hours) or more frequent (4 sessions per week) hemodialysis regimens versus remaining on 3 sessions per week with session lengths of 4 hours.

**Analytical Approach:**

Multinomial mixed effects logistic regression estimating the relative influence of different levels of the predictors on the selection of longer and more frequent dialysis, controlling for patient demographic characteristics.

**Results:**

Among 183 prevalent in-center hemodialysis patients (mean age of 63.7 years, mean dialysis vintage of 4.7 years), 38.3% (70 of 183) always chose to remain on regimens of 3 sessions per week with session duration of 4 hours. Depicted associations of increasing survival and quality of life, reduced need for fluid restriction, and avoiding additional access complications were all significantly associated with choosing longer or more frequent treatment regimens. Younger age, fatigue, previous experience of vascular access complications, absence of heart failure, and shorter travel time to dialysis centers were associated with preference for 4 sessions per week. Patients expressed willingness to trade up to 2 years of life to avoid regimens of 4 sessions per week or access complications. After applying estimated treatment benefits and harms from existing literature, the fully adjusted model revealed that 27.1% would choose longer regimens delivered 3 times per week and 34.3% would choose 4 hours 4 times per week. Analogous estimates for younger fatigued patients living near their unit were 23.5% and 62.5%, respectively.

**Limitations:**

Estimates were based on stated preferences rather than observed behaviors. Predicted acceptance of regimens was derived from data on treatment benefits and harms largely sourced from observational studies.

**Conclusions:**

Predicted acceptance of longer and more frequent hemodialysis regimens substantially exceeds their use in current clinical practice. These findings underscore the need for robust data on clinical effectiveness of these more intensive regimens and more extensive consideration of patient choice in the selection of dialysis regimens.

**Plain-Language Summary**

Longer or 4-times-a-week dialysis has been associated with better outcomes, yet their use is limited, and they are perceived as undesirable to patients. One hundred and eighty-four people on dialysis completed a discrete choice questionnaire that presented the association of these longer and more frequent treatments with longer survival, less hospitalization, better quality of life, and fewer vascular access complications. Presented with available evidence on these associations, 27.1% of patients would choose longer dialysis, and 34.3% would choose 4-times-per-week dialysis, far more than is currently observed in routine practice. Better data on clinical effectiveness to guide patient choice are needed.

Editorial, p. 778

Observational and clinical trial data have shown survival and quality of life advantages for more intensive hemodialysis (HD) regimens than the 4-hour 3-times-per-week regimens recommended by clinical practice guidelines.^1^ These regimens include longer session length delivered 3 times per week, and 4 sessions per week. Despite the stated advantages, acceptance of these treatments in routine clinical practice and clinical trials suggests that underlying patient preferences and treatment burden may be factors influencing a patient’s choice.^2^

Through a range of potential mechanisms, more intensive dialysis regimens have potential benefits but also potential harms; these effects include fatigue, survival, cardiovascular disease, and vascular access patency, which have been identified as core HD trial outcomes in consensus exercises.^3^, ^4^, ^5^ Previous stated preference work has reported on the proportion of patients who might select more intensive HD regimens, but not in the presence of outcome information, which should ideally be presented as part of shared decision making, tailored to the characteristics and goals of the individual.^6^^,^^7^

By eliciting preferences to the possible benefits and harms of a treatment, the relative importance of individual trial end points for an intervention can be identified.^8^^,^^9^ In addition to providing further clarity around clinically meaningful differences, the potential size of the benefit required to meaningfully change acceptance of a treatment can be estimated.^10^ A comprehensive understanding of patient preferences could assist in predicting capacity requirements and identify groups who need additional education or support during trial recruiting or when undertaking shared decisions around treatment.

Discrete choice experiments (DCEs) have been shown to accurately estimate patient preferences toward treatments by asking patients to consider treatment options while the potential benefits and harms of the different treatment options are varied.^11^ We present a DCE conducted across 5 centers in the United Kingdom designed to elicit patient preferences toward longer session length, more frequent HD, or remaining on the standard regimen, all delivered in-center.^12^^,^^13^ Accounting for individual patient characteristics that influence these preferences, we estimated the acceptance of these more intensive regimens in the presence of benefits and harms identified in the literature for a prevalent population and specific groups.

## Methods

This labeled DCE is reported in accordance with ISPOR (International Society for Pharmacoeconomics and Outcomes Research) good research practice recommendations.^14^^,^^15^ The DCE was designed to elicit preferences toward the dialysis regimen choices of longer session length (delivered 3 times per week), versus more frequent sessions (4 times per week), versus remaining on 3-times-per-week hemodialysis, with the preferences dependent on 4 outcome-based treatment attributes: survival, hospitalization, quality of life, and vascular access complications. By asking the respondent to complete multiple-choice sets, the relative importance of the attributes, their levels, the choices (dialysis regimens), and any trade-offs could be estimated. Detailed information for the discrete-choice experiment methodology applied in health care can be found elsewhere.^15^ Ethical approval for the study was obtained in June 2019 (Health Research Authority IRAS reference 253384), and the participants were recruited between February 2019 and November 2019.

### Participants and Study Perspective

The inclusion criteria for the study were prevalent in-center HD patients who had been receiving treatment for at least 1 year. This ensured sufficient experience of in-center HD to consider and relate to the scenario and aligned with the informing studies in which the majority of patients who received these treatments had been dialyzing in excess of 1 year.^12^^,^^13^ The exclusion criteria were an existing diagnosis of malignancy (because patients with a limited life expectancy may not be offered these regimens and may have their treatment shortened toward the end of life),^12^ or the presence of a formal diagnosis of cognitive impairment in the medical notes, or the presence of cognitive impairment as assessed by the dialysis nursing staff or the researcher conducting the questionnaire.

The questionnaire booklet begins by asking participants to consider a scenario where they were an in-center HD patient who after 2 years of treatment had developed high ultrafiltration rates and significant fatigue. In the scenario, the staff at the dialysis unit hypothetically offer them these treatments: longer sessions (4.5 hours) provided 3 times per week,^12^ more frequent (4 times per week) 4-hour duration HD,^13^ or remaining on the current 4 hours provided 3 times per week (an opt-out choice). The questionnaire then asks the participant to consider which HD regimen they would select in this scenario 12 times. With each of the 12 questions (choice sets) the associated benefits and harms (survival, hospitalization, quality of life, fluid restriction, and vascular access complications) varied in a prespecified manner to build a statistical model of the individual’s preferences (Item S1).

### Treatment Choices, Attributes, and Levels

We presented 2 more-intensive regimen choices that were based on published data evaluating these treatments in the context of the increased hospitalization and mortality associated with the long interdialytic interval intrinsic to 3-times-per-week HD schedules,^16^ and that were designed to be plausible and available in existing clinical practice. Attributes had all been prioritized in existing mixed methods research. In order to predict acceptance, existing evidence between attributes and the treatment regimens being offered informed the attribute and level selection.^7^^,^^12^^,^^13^^,^^17^^,^^18^ Each of the 12 DCE questions included a labeled description of longer, 4-times-a-week, and continuing 3-times-a-week HD and the attributes associated with them for that particular DCE choice set. Table 1 shows an example of the attributes presented to the patient for the third DCE choice set, which illustrates the range of levels each attribute could take: survival (9, 10, 12, or 14 years), quality of life (you feel the same; you feel better), fluid restriction (you can drink the same; you can drink more), hospitalization (once a year; once every 2 years), and access complications (no change; more complications). All 12 DCE choice sets are presented in Item S1.

**Table 1:** Example of Discrete Choice Experiment Choice Set

	Longer Sessions	Extra Session	No Change
**Description**			
Frequency	3 times a week	4 times a week	3 times a week
Session length	4.5 hours	4 hours	4 hours
**Information**			
Survival	10 years	12 years	9 years
Quality of life	You feel the same	You feel better	You feel the same
Fluid restriction	You can drink the same	You can drink more	You can drink the same
Hospitalization	Once a year	Once every 2 years	Once a year
Access complications	More complications	No change	No change

Shown is choice set number 3; different attribute levels (eg, 14-year survival) were presented in other choice sets. Respondents were asked to check a box corresponding to the dialysis regimen they would select.

In order to present absolute years of survival for the survival attribute, the survival associated with continuing on standard HD for 9 years was estimated using a parametric exponential survival model fitted on the patients with ultrafiltration rates of >10 mL/kg/h who continued to receive 3-times-per-week HD in an informing analysis.^13^ This model included age, sex, comorbidities, phosphate, dialysis access, and ultrafiltration rate as adjustment variables.

### Instrument Design and Sample Size

The goal of the DCE is to build a model from which the relative influence of each of the attributes and anything pertaining to the treatments themselves can be estimated. Undertaking this could involve presenting every permutation of the attributes and asking the respondent to select a treatment, but this is rarely practical. Fewer DCE questions may result in improved response efficiency (the measurement error associated with respondent inattention introduced by too many questions).^14^ It is considered common practice to have between 8 and 16 DCE questions,^15^ with reviews highlighting 70% of studies having 3 to 6 attributes with up to 4 attribute levels.^19^ A full-choice array containing every possible permutation of the attribute levels was generated and from this a D-efficient design was identified by sampling subsets of this array. This was performed using the DCREATE command in STATA^20^ resulting in a randomly ordered design with 12 DCE questions and a D-efficiency of 1.607. A sample size of 128 respondents for the 12 question DCE was estimated with the approximate formula^21^ using an α of 0.95, accuracy of 10%, and an expected choice proportion of 20%.^6^ This was doubled to 256 to allow for subgroup and interaction effects estimation.

### Data Collection

Research nurses and clinical trial assistants screened individuals based on the inclusion criteria then approached HD patients on the dialysis unit for consent to perform the paper questionnaire. Often the patient would complete the questionnaire while receiving dialysis and with the researcher nearby facilitating assistance when required, in line with ISPOR good research practices.^14^^,^^15^ After an explanation of the decision scenario, the respondent undertook a comprehension question that presented the treatments with hypothetical benefits and harms in the same format as the rest of the DCE, which asked the respondent to state which treatment has the best levels for each of the 5 attributes. The 12 DCE questions were then completed along with some demographic information including the SONG-HD (Standardized Outcomes in Nephrology–Hemodialysis) fatigue measure,^22^ travel time to the dialysis unit, personal experience of HD access problems, whether the more intensive regimens had previously been offered, and a short health literacy question.^23^ The researchers completed a demographic information from patients notes including comorbidities, HD schedule, dialysis access, hemoglobin, and ultrafiltration volume.

### Statistical Analysis

The differences in patient characteristics according to whether the patient had been previously offered more intensive dialysis were statistically assessed using independent *t* tests for continuous variables and χ^2^ tests for categorical variables.

A multinomial logistic model with random coefficients (mixed effects), with the selection of one of the treatments as the dependent (outcome) variable was used to estimate the relative influence of different levels of the attributes and description of the treatment regimens, with odds ratios reported.^24^ A mixed model allows for correlated preferences (eg, a patient having a greater preference for both longer and more frequent dialysis) and is fit on data with an observation per treatment offered (eg, 3 observations per choice set). Allowing the constants associated with the descriptions of the treatments to vary between respondents provided a superior fit compared with fixed values for all respondents, and the standard deviation of all random parameters was significant. The final models were estimated with 1,000 Halton draws.

Patient characteristics that could influence preferences for attributes and choices were controlled for by specifying interactions between these variables in the model. All choice attributes were treated as categorical with the demographic variables of age (<50, 50-80, and >80 years), travel time (<30 or ≥30 minutes), and time on kidney replacement therapy (<2, 2-5, and >5 years) categorized based on their distributions. The SONG-HD fatigue measure was calculated based on the sum of questions on feeling tired, lacking energy, and limits on usual activities individually scored 0-3 (total score of 0-9).^22^ All analyses including those with interactions use the opt-out of continuing on a regimen of 3 times per week, 4 hours as the reference.

The best-performing model in the presence of interactions was identified using Akaike information criterion, which penalizes for additional covariates. Using the model with the best performance, trade-offs between survival and other attributes and the predicted acceptance of treatments were estimated. Survival in years was treated as a continuous variable in the model, and using the STATA WTP^25^ command an estimate of the number of years patients would sacrifice to improve other attributes or avoid the treatment burden associated with the more intensive regimens was calculated. Because individuals who always choose the opt-out (to stay on 3-times-per-week dialysis for 4 hours) have an infinitely large choice specific constant for the opt-out alternative—potentially resulting in bias—the results of the analysis are presented only in those who always chose the opt-out excluded. An analysis of all patients is reported in Table S1.

Interactions between patient characteristics and choice-specific constants were specified as fixed effects in all models. The probability of acceptance of the regimens was estimated using the model with the best Akaike information criterion: a systematic review that informed UK HD guidelines was updated and effect sizes for the treatment attributes associated with the different regimens extracted.^1^ Applying these effect sizes to estimates of survival, quality of life, fluid restriction, and access complications to informing literature and the parametric survival estimate identified the values to set the attributes for each regimen. Acceptance of treatments is reported for the cohort who completed the questionnaire, for patients with specific characteristics determined by those included in the informing clinical trials,^4^ or for clinically relevant subgroups. The attributes associated from the literature (largely observational) alongside more conservative estimates, informed by a reduction of 1 level of any attributes associated with improved outcomes, are detailed in Table 5.

## Results

Across 5 centers, 292 patients were approached, of whom 204 consented (69.9%); 196 patients returned the questionnaire, and 183 completed all 12 DCE questions. The demographics were comparable to prevalent in-center HD patients in the United Kingdom and observational studies informing the questionnaire. After reading the description of the fictional patient in the opening scenario, 40.2% (47 of 117) of respondents felt it sounded like them, and a further 38.5% (45 of 117) thought it sounded somewhat like them.

Table 2 reports the overall demographics of those completing the questionnaire, which is then stratified by whether more intensive dialysis had (65/183, 35.5%) or had not (118/183, 64.5%) been discussed. Overall, 24.7% and 14.8% of patients had previously been approached about longer hours and more frequent HD, respectively. Patients who had been offered more intensive dialysis were statistically more likely to be younger, male, live nearer the renal center, and have had previous dialysis access complications, with a tendency to have more comorbidities. Feeling that the scenario sounded like them was associated with a higher fatigue score (4.9 vs 3.1, *P* = 0.002). Overall, 53.6% (97/181) had previously experienced vascular access problems.

**Table 2:** Patient Demographics

	Overall	Previously Offered More Intensive Dialysis		
		Yes (Longer and/or 4×/wk)	No	*P*
No. of patients	183	65/183	118/183	
Age, y	63.7 ± 15.4	60.1 ± 16.0	65.7 ± 14.7	0.009
<50 y	18.6% (34/183)	23% (15/65)	16.1% (19/118)	
50-80 y	67.2% (123/183)	68% (44/65)	66.9% (79/118)	
>80 y	14.2% (26/183)	9% (6/65)	17.0% (20/118)	
Male sex	63.4% (116/183)	77% (50/65)	55.9% (66/118)	0.005
White ethnicity	80.3% (147/183)	79% (51/65)	81.4% (96/118)	
Comorbidity				
Diabetes	36.6% (67/183)	42% (27/65)	33.9% (40/118)	0.3
Previous MI	9.3% (17/183)	11% (7/65)	8.5% (10/118)	0.6
Heart failure	10.4% (19/183)	15% (10/65)	7.6% (9/118)	0.1
Weight, kg	83.4 ± 25.4	85.3 ± 25.3	82.4 ± 25.5	0.8
Ultrafiltration				
2-day interval, mL/kg/h	6.8 ± 3.2	6.6 ± 3.4	6.9 ± 3.1	0.3
1-day interval, mL/kg/h	5.2 ± 2.8	5.3 ± 2.8	5.2 ± 2.9	0.6
Hemoglobin, g/L	106.3 ± 19.7	108.6 ± 16.2	105.1 ± 21.5	0.9
Time on dialysis, y	4.3 ± 4.2	4.0 ± 3.4	4.5 ± 4.6	0.2
<2 y	30.6% (56/186)	28% (18/65)	32.2% (38/118)	
2-5 y	41.5% (76/186)	51% (33/65)	36.2% (43/118)	
>5 y	27.4% (51/186)	22% (14/65)	31.4% (37/118)	
Dialysis access				0.4
AVF	72.6% (130/179)	72% (46/64)	73.0% (84/115)	
Catheter	23.5% (42/179)	25% (16/64)	22.6% (26/115)	
Other	3.9% (7/179)	3% (2/64)	4.4% (5/115)	
Mon/Wed/Fri schedule	56.1% (101/180)	66% (42/64)	50.9% (59/116)	0.1
SONG-HD Fatigue score	4.9 ± 2.5	5.1 ± 2.7	4.8 ± 2.4	0.2
<4	35.0% (64/183)	34% (22/65)	35.6% (42/118)	
4-7	44.8% (82/183)	45% (29/65)	44.9% (53/118)	
>7	20.2% (37/183)	22% (14/65)	19.5% (23/118)	
Previous access complications	53.6% (97/181)	68% (43/63)	45.8% (54/118)	0.004
Dialysis travel time, min	25.0 ± 16.4	20.8 ± 14.0	27.3 ± 17.2	0.005
Inadequate health literacy	15.6% (28/180)	19% (12/50)	13.6% (16/118)	0.3

Values for continuous variables given as mean ± standard deviation. Abbreviations: AVF, arteriovenous fistula; MI, myocardial infarction; SONG-HD, Standardized Outcomes in Nephrology–Hemodialysis.

For the comprehension test, 23.3% (42 of 180) of patients incorrectly answered all 5 questions, which did not significantly vary with health literacy (*P* = 0.8).

### Treatment and Outcome Preferences

From 183 completed 12-question DCEs resulting in 2,196 choices, longer (4.5-hour) dialysis sessions were selected by 29.3%, more frequent (4 times per week) by 20.4%, and continuing on 3-times-per-week HD by 50.3%. Increasing quality of life and survival and reduced fluid restriction with a regimen all had a clinically plausible, positive influence on the selection of a more intensive regimen whereas increased vascular access complications associated with a regimen reduced the likelihood of a regimen’s selection (Table 3). Hospitalization had no influence. The adjusted odds ratios (eg, benefits and harms set to that of 3-times-per-week HD) were 0.06 (95% CI, 0.02-0.14) for selection of longer hours and 0.005 (95% CI, 0.001-0.01) for more frequent HD. These adjusted values in isolation are only illustrative because patients and clinicians consider offering or accepting these regimens in the presence of benefits and harms that are generally more preferable for the revised treatment being offered, rather than the same as the current treatment. These estimates did not significantly differ when limited to the individuals who got none of the comprehension test questions wrong (Table S2).

**Table 3:** Adjusted Odds Ratios From Multivariable Analysis for the Selection of Longer and More Frequent Dialysis, Presented Alongside Their Potential Benefits and Harms

	Coefficient	Odds Ratio (95% CI)	*P*
Survival			
10 y (+1 y)	1.01 (0.53, 1.48)	2.73 (1.7-4.39)	<0.001
12 y (+2 y)	3.24 (2.64, 3.84)	25.50 (13.97-46.55)	<0.001
14 y (+4 y)	3.79 (3.08, 4.51)	44.36 (21.7-90.7)	<0.001
Quality of life improved	0.40 (0.04, 0.76)	1.49 (1.04-2.14)	0.03
Fluid restriction relaxed	0.47 (0.12, 0.83)	1.61 (1.13-2.28)	0.008
Hospitalization reduced	0.11 (−0.15, 0.38)	1.12 (0.86-1.46)	0.4
Access complications increased	−2.12 (−2.63, −1.62)	0.12 (0.07-0.2)	<0.001
Longer (4.5 h, 3×/wk)	−2.86 (−3.75, −1.97)	0.06 (0.02-0.14)	<0.001
More frequent (4×/wk)	−5.39 (−6.54, −4.24)	0.005 (0.001-0.01)	<0.001

Multivariable adjusted coefficients and odds ratios for dialysis regimens represent the likelihood of being selected if there were no benefits or harms compared with 3×/wk, 4-h hemodialysis. The overall likelihood of a treatment being selected can be estimated by the sum of the coefficients for a given treatment. For instance, for 4×/wk resulting in +2-y survival, quality of life improved, and fluid restriction relaxed: 3.24 + 0.40 + 0.47 − 5.39 = −0.02.

### Interactions Among Demography, Experience, Symptoms, and Choice

**Figure 1:**
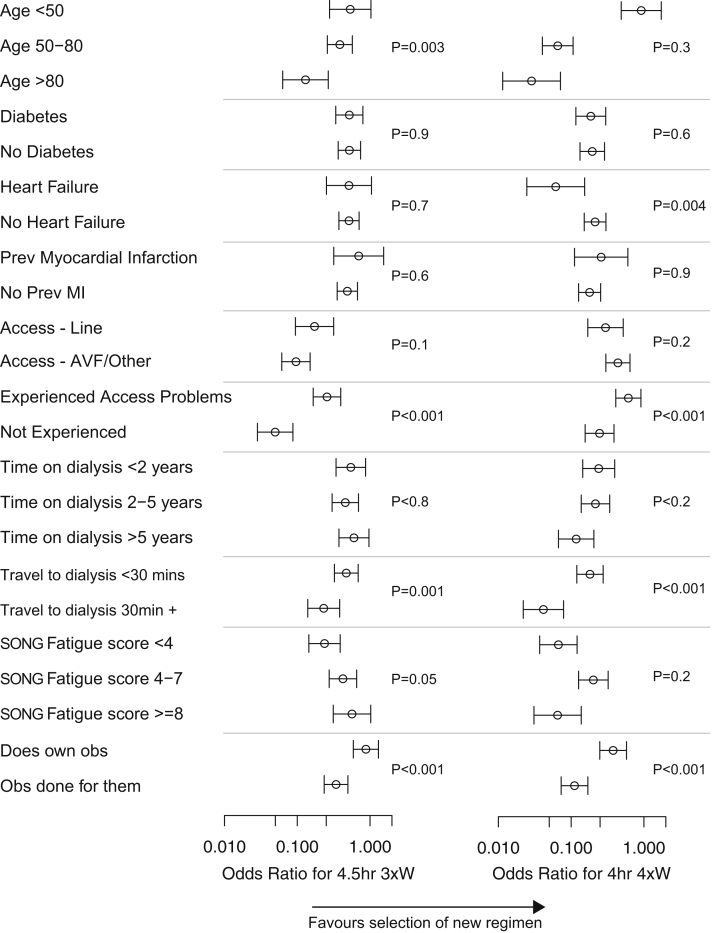
Treatment-specific constant interactions. Abbreviations: AVF, arteriovenous fistula; MI, myocardial infarction; obs, observations; Prev, previous; SONG, Standardized Outcomes in Nephrology.

**Figure 2:**
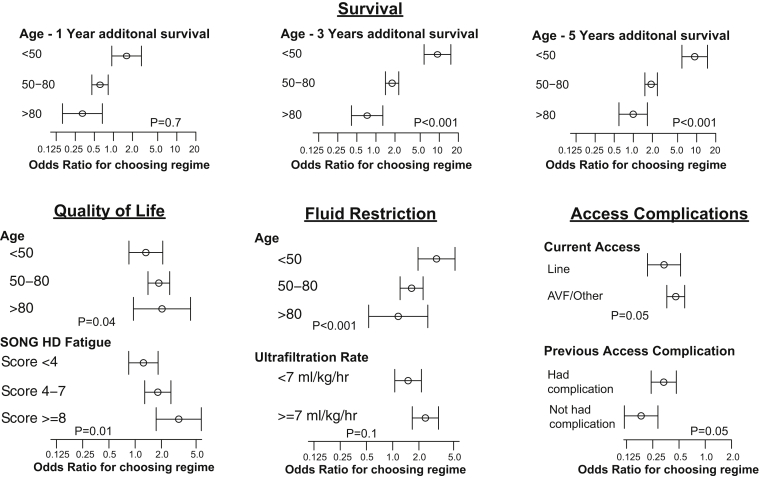
Attributes and interactions. Abbreviations: AVF, arteriovenous fistula; SONG-HD, Standardized Outcomes in Nephrology–Hemodialysis.

Patients under the age of 50 had a stronger preference for the more intensive regimens, and decreasing age was associated with significant increases in how patients valued survival advantages and fluid restrictions (Figs 1 and 2). Higher ultrafiltration rates did not modify preferences toward reduced fluid restriction (Fig 2).

The patients who had experienced a vascular access complication found both regimens more acceptable than those who had not (Fig 1). Having experienced a dialysis access complication or receiving dialysis via a fistula rather than a catheter/line was associated with greater preference for more-intensive regimens. The absence of heart failure significantly increased the preference for 4-times-per-week HD, but diabetes and previous myocardial infarction had no influence on treatment preference. There was no interaction between comorbidities and the attributes of survival or quality of life.

Patients who scored less than 4 on the SONG-HD Fatigue measure had a very low preference toward 4-times-per-week HD. Increasing fatigue was associated with increasing preference toward quality of life improvements associated with a regimen, particularly in patients scoring >7. There was no relationship between higher health literacy, Tuesday/Thursday/Saturday dialysis schedule compared to the Monday/Wednesday/Friday schedule, or number of years treated by HD toward either more intensive regimen.

### Trading Survival for Improvements in Other Attributes

The fully interacted model (Table S3) resulted in linear increases in the coefficients informing the odds ratios for increasing survival in years, enabling the estimation of trade-offs between survival and the other attributes. The 38.3% (70/183) who always chose to stay on 3-times-per-week 4-hour HD introduced a bias in the estimates due to having an infinitely small treatment-specific odds ratio, and after their exclusion the following estimates were obtained (Table 4, model reported in Table S3): patients would sacrifice approximately 2 years of life to avoid attending 4 times per week or having an access complication, and they would sacrifice more liberal fluid intake or quality of life for an additional two-thirds of a year of survival.

**Table 4:** Years of Patient Survival Traded for Improvements in Other Attributes or Avoiding Longer/More Frequent Dialysis

	Years of Survival Traded (95% CI)
Longer (4.5 h, 3×/wk)	−1.03 (−2.43 to 0.36)
More frequent (4×/wk)	1.98 (0.29 to 3.67)
Quality of life	−0.65 (−1.11 to −0.20)
Fluid restriction	−0.67 (−1.13 to −0.20)
Access complication	2.23 (1.49 to 2.97)

Negative values: in the absence of other attributes or change in hemodialysis regimen (due to multivariable adjustment), the number of years of survival a patient would give to obtain the attribute. Positive values: the number of years of survival a patient would give to avoid the attribute. The 70 of 183 respondents who always chose to stay on 4-hour 3-times-per-week hemodialysis are excluded.

### Projected Acceptance of Longer or More Frequent Hemodialysis

Patient characteristics, hypothesized treatment benefits, and model specification influenced the proportion of patients who would choose longer dialysis, more frequent dialysis, or opt to remain on their current treatment (Table 5). Based on the more optimistic treatment benefits, 29.1% would dialyze for 4.5 hours, 35.8% would dialyze 4 times per week, and 35.1% would remain on their current treatment, with these proportions changing to 27.1%, 34.3%, and 38.6%, respectively, when incorporating patient characteristics in the model. More conservative benefits generally increased the percentage opting to remain on the current treatment by approximately 10 percentage points. Simulating a cohort comparable with those recruited into the Frequent Hemodialysis Network (FHN) trial, the proportion selecting 4-times-per-week HD was 44.6%. Elderly patients with moderate fatigue who live far from the unit would select 4.5-hour and 4-times-per-week HD 11.0% and 22.0% of the time, and the proportions for younger severely fatigued patients living nearer the unit were 23.5% and 62.5%, respectively.

**Table 5:** Probability of Acceptance According to Patient Characteristic and Available Evidence

Cohort	Patient/Treatment Characteristics	Standard Estimates			Conservative Estimates		
		3×/wk, 4.5 h	4×/wk, 4 h	Opt Out	3×/wk, 4.5 h	4×/wk, 4 h	Opt Out
		+1 y Survival,^12^ No Other Benefits^31^	+2 y Survival,^13^ ↓ Fluid Restriction,^4^ ↑ QoL^3^	No Change in Attributes	No Change in Attributes	+1 y Survival	No Change in Attributes
Sampled population	See Table 2, interactions specified^a^	0.271 (0.254-0.289)	0.343 (0.324-0.361)	0.386 (0.370-0.402)	0.239 (0.219-0.259)	0.272 (0.256-0.287)	0.489 (0.470-0.508)
FHN Trial	Age 50 ± 14 y Fatigue score 4.7 ± 2.2 20% heart disease 39% on HD for 2-5 y 42.7% do own obs 65% live near the unit	0.259 (0.246-0.272)	0.446 (0.434-0.458)	0.295 (0.289-0.302)	0.233 (0.219-0.247)	0.371 (0.357-0.386)	0.396 (0.382-0.410)
Established, elderly patient	Age 82 y Moderate fatigue Lives far from the unit	0.110 (0.073-0.147)	0.220 (0.207-0.232)	0.670 (0.645-0.696)	0.078 (0.038-0.119)	0.147 (0.139-0.155)	0.774 (0.742-0.807)
Young, working-age patient	Age 45 y Severe fatigue Does own obs Lives near the unit	0.235 (0.218-0.251)	0.625 (0.608-0.642)	0.140 (0.133-0.147)	0.230 (0.210-0.251)	0.554 (0.537-0.572)	0.215 (0.205-0.226)

Values reported are the probability of acceptance of the new regimen (95% CI). Age and fatigue (based on visual analog scale) score given as mean ± SD. Abbreviations: FHN, Frequent Hemodialysis Network; HD, hemodialysis; obs, observations; QoL, quality of life.

a Interactions specified: choice-specific constants and the variables of age, time on dialysis, heart failure, fatigue, undertaking own observations, and travel time.

## Discussion

This multicenter study used a DCE to estimate preferences toward the benefits and harms associated with longer and more frequent in-center HD regimens. Improvements in quality of life, survival, and fluid restriction were associated with selecting a more intensive regimen. Younger, more fatigued patients who were able to do their own blood pressure, pulse, and temperature while undergoing HD (and perhaps other dialysis-related tasks) were more likely to choose 4-times-per-week dialysis. However, longer and more frequent HD could be considered undesirable because 38.5% of patients completing the DCE did not choose them in any situation, and those who did would still sacrifice 2 years of additional life to avoid them. Despite this, if presented with benefits of these regimens from the literature, between half and two-thirds of patients would be willing to be treated with dialysis regimens that are 4 times per week or to undergo HD for longer than 4 hours 3 times per week.

Our findings corroborate existing research: 33.5% of US HD patients who were struggling with their fluid restriction said they would dialyze for an additional 30 minutes, and 19.6% would do an additional weekly session, although the benefits associated with these regimens were not presented. Patients from the US study were generally closer to the patient phenotype described in our scenario and in whom these interventions are routinely used.^6^ A study using conjoint analysis identified that 44% of sampled patients would not select daily HD irrespective of the potential health benefits; however, 38.9% of patients would choose the treatment if the quality-of-life and survival benefits were comparable to those applied to our study’s 4-times-per-week regimen.^26^ Both studies found greater acceptance in younger, less comorbid patients.

The statistical and clinical significance of the attributes of survival, quality of life, and avoiding vascular access complications, with the lower importance of hospitalization, aligns with recent prioritization exercises for clinical trial end points in HD.^18^ Direct comparison of the predicted acceptance of treatments in our cohort to other studies is challenging: simulating the FHN daily trial cohort who were offered 5-6 sessions a week, 37% to 44% of patients would select 4-times-per-week HD compared with the 12% of those approached who had agreed to participate in the FHN trial. A third of patients in our study had been approached about more intensive regimens, with real-world data suggesting around 3.5% would subsequently receive 4-times-per-week HD and 18% would receive 4.5-hour 3-times-per-week HD.^12^^,^^13^ Discrepancies between real-world use and predicted uptake could relate to observed and unobserved differences in the cohorts approached, patient interpretation of the choice scenario, or the statistical models.

To tackle the long interdialytic interval, the fourth session should ideally be scheduled during this period, although some patients may wish to preserve a 2-day gap. However, patients can recognize and quantify the potential survival and quality of life benefits associated with an additional session during the traditional long interdialytic interval.^27^ More generally, models from DCE studies have been shown to have reasonable positive predictive value for choices made in real-world clinical practice,^28^ but the disproportionate presence of selecting the status quo exists both in this study and many others exploring decision making.^29^

The strengths of our study include a strong underlying methodological design to elicit preferences and the presentation of the HD regimens that are currently available and for which some estimates of efficacy are known. The presented scenario resonated with 78.6% of respondents, all of whom had personal experience of HD. The modest sample size exceeded formal power calculations, and where possible the estimates on subgroups were drawn from interactions, retaining the overall sample size. The weaknesses included the assessment of stated preferences and not genuine choices that the patient subsequently made, potentially overoptimistic baseline survival estimates for the scenario, and acceptance probabilities being informed by largely observational data. A quarter of patients answered all 4 comprehension test questions incorrectly, which may relate to questionnaire complexity or the cognitive function of the patient group.

Findings from our study raise the policy and future research issue that if patient acceptance of these treatments is as high as estimated in our study, it is even more important to obtain high-quality evidence to determine their clinical effectiveness before more routine presentation of these regimens is made to patients. The difference between predicted and observed uptake of these treatments suggests that some individuals may be willing to do more HD treatment to access the benefits reported in the literature, but only a third of this cohort had actually been approached regarding the treatment options. The findings do allude to certain groups of patients who are more likely to accept these treatments, which could inform models of HD capacity. Significant increases in uptake could be offset by an incremental approach to HD dosing that would include lower frequency when starting dialysis.^30^

Integral to these decisions around treatment would be the presentation of information obtained from generalizable trials of these interventions in HD patients, for which it has been challenging to recruit or retain patients or to statistically demonstrate health benefits.^2^^,^^31^^,^^32^ This person-centered shared decision making would need to elicit the treatment goals of the patient as prioritized in other settings, to consider whether in-center HD could deliver these, and to offer alternatives.^7^^,^^33^ Based on the findings from our study, there is an increasing imperative to gain high-quality data on the clinical and cost-effectiveness of these treatment options to advocate for their use with decision-makers and to inform patients in whom the treatments are indicated.

## Supplementary Material

Supplementary File (PDF) Item S1. Tables S1-S3.
