# Supplementary material for: Patient Preferences for Longer or More Frequent In-Center Hemodialysis Regimens: A Multicenter Discrete Choice Study
Source: Am J Kidney Dis. 2022 Jun;79(6):785–95. doi: 10.1053/j.ajkd.2021.09.012 (PMC9153730; doi:10.1053/j.ajkd.2021.09.012)
Supplement: Supplementary File (PDF) — Item S1. Tables S1-S3. [file mmc1.pdf]

Item S1 – Summary of the Discrete Choice Experiment Choice Sets

Question 1 of 12

Description	Longer sessions	Extra Session	No Change
Frequency	Three times a week	Four times a week	Three times a week
Session length	4 and a half hours	4 hours	4 hours
**Information**			
Survival	9 years	12 years	9 years
Quality of Life	You feel better	You feel the same	You feel the same
Fluid Restriction	You can drink more	You can drink the same	You can drink the same
Hospitalisation	once every two years	once a year	once a year
Access Complications	No change	More complications	No change

Question 2 of 12

Description	Longer sessions	Extra Session	No Change
Frequency	Three times a week	Four times a week	Three times a week
Session length	4 and a half hours	4 hours	4 hours
**Information**			
Survival	14 years	9 years	9 years
Quality of Life	You feel the same	You feel better	You feel the same
Fluid Restriction	You can drink the same	You can drink more	You can drink the same
Hospitalisation	once a year	once every two years	once a year
Access Complications	No change	More complications	No change

Question 3 of 12

Description	Longer sessions	Extra Session	No Change
Frequency	Three times a week	Four times a week	Three times a week
Session length	4 and a half hours	4 hours	4 hours
**Information**			
Survival	10 years	12 years	9 years
Quality of Life	You feel the same	You feel better	You feel the same
Fluid Restriction	You can drink the same	You can drink more	You can drink the same
Hospitalisation	once a year	once every two years	once a year
Access Complications	More complications	No change	No change

Question 4 of 12

**Description**	**Longer sessions**	**Extra Session**	**No Change**
Frequency	Three times a week	Four times a week	Three times a week
Session length	4 and a half hours	4 hours	4 hours
**Information**			
Survival	12 years	14 years	9 years
Quality of Life	You feel better	You feel the same	You feel the same
Fluid Restriction	You can drink the same	You can drink more	You can drink the same
Hospitalisation	once every two years	once a year	once a year
Access Complications	More complications	No change	No change

Question 5 of 12

**Description**	**Longer sessions**	**Extra Session**	**No Change**
Frequency	Three times a week	Four times a week	Three times a week
Session length	4 and a half hours	4 hours	4 hours
**Information**			
Survival	9 years	14 years	9 years
Quality of Life	You feel the same	You feel better	You feel the same
Fluid Restriction	You can drink more	You can drink the same	You can drink the same
Hospitalisation	once a year	once every two years	once a year
Access Complications	More complications	No change	No change

Question 6 of 12

**Description**	**Longer sessions**	**Extra Session**	**No Change**
Frequency	Three times a week	Four times a week	Three times a week
Session length	4 and a half hours	4 hours	4 hours
**Information**			
Survival	12 years	10 years	9 years
Quality of Life	You feel the same	You feel better	You feel the same
Fluid Restriction	You can drink the same	You can drink more	You can drink the same
Hospitalisation	once every two years	once a year	once a year
Access Complications	No change	More complications	No change

Question 7 of 12

**Description**	**Longer sessions**	**Extra Session**	**No Change**
Frequency	Three times a week	Four times a week	Three times a week
Session length	4 and a half hours	4 hours	4 hours
**Information**			
Survival	10 years	12 years	9 years
Quality of Life	You feel better	You feel the same	You feel the same
Fluid Restriction	You can drink the same	You can drink more	You can drink the same
Hospitalisation	once every two years	once a year	once a year
Access Complications	More complications	No change	No change

Question 8 of 12

**Description**	**Longer sessions**	**Extra Session**	**No Change**
Frequency	Three times a week	Four times a week	Three times a week
Session length	4 and a half hours	4 hours	4 hours
**Information**			
Survival	14 years	10 years	9 years
Quality of Life	You feel the same	You feel better	You feel the same
Fluid Restriction	You can drink more	You can drink the same	You can drink the same
Hospitalisation	once every two years	once a year	once a year
Access Complications	More complications	No change	No change

Question 9 of 12

**Description**	**Longer sessions**	**Extra Session**	**No Change**
Frequency	Three times a week	Four times a week	Three times a week
Session length	4 and a half hours	4 hours	4 hours
**Information**			
Survival	14 years	9 years	9 years
Quality of Life	You feel better	You feel the same	You feel the same
Fluid Restriction	You can drink more	You can drink the same	You can drink the same
Hospitalisation	once a year	once every two years	once a year
Access Complications	More complications	No change	No change

Question 10 of 12

**Description**	**Longer sessions**	**Extra Session**	**No Change**
Frequency	Three times a week	Four times a week	Three times a week
Session length	4 and a half hours	4 hours	4 hours
**Information**			
Survival	10 years	14 years	9 years
Quality of Life	You feel the same	You feel better	You feel the same
Fluid Restriction	You can drink more	You can drink the same	You can drink the same
Hospitalisation	once every two years	once a year	once a year
Access Complications	No change	More complications	No change

Question 11 of 12

**Description**	**Longer sessions**	**Extra Session**	**No Change**
Frequency	Three times a week	Four times a week	Three times a week
Session length	4 and a half hours	4 hours	4 hours
**Information**			
Survival	12 years	9 years	9 years
Quality of Life	You feel better	You feel the same	You feel the same
Fluid Restriction	You can drink more	You can drink the same	You can drink the same
Hospitalisation	once a year	once every two years	once a year
Access Complications	No change	More complications	No change

Question 12 of 12

**Description**	**Longer sessions**	**Extra Session**	**No Change**
Frequency	Three times a week	Four times a week	Three times a week
Session length	4 and a half hours	4 hours	4 hours
**Information**			
Survival	9 years	10 years	9 years
Quality of Life	You feel better	You feel the same	You feel the same
Fluid Restriction	You can drink the same	You can drink more	You can drink the same
Hospitalisation	once a year	once every two years	once a year
Access Complications	No change	More complications	No change

Table S1: Non-interacted model output with survival as a continuous variable, overall and in those did not always choose the opt-out

	All Patients		Excluding those who always chose opt-out	
	Coefficient (95% CI)	P	Coefficient (95% CI)	P
Longer	-1.72 (-2.34 to -1.1)	<0.001	0.39 (-0.08 to 0.86)	0.106
4xW	-4.97 (-6.06 to -3.87)	<0.001	-1.92 (-2.8 to -1.03)	<0.001
Survival (per year)	0.38 (0.22 to 0.55)	<0.001	0.77 (0.58 to 0.97)	<0.001
Fluid Restriction	0.36 (-0.02 to 0.74)	0.061	0.52 (0.15 to 0.9)	0.006
Quality of Life	0.36 (-0.03 to 0.75)	0.073	0.52 (0.12 to 0.91)	0.01
Vascular Access Complications	-2.09 (-2.58 to -1.6)	<0.001	-1.82 (-2.39 to -1.24)	<0.001

Table S2: Non-interacted model output in those who answer all cognition questions correctly

Mixed logit model	Number of obs	=	5,004
	LR chi2(9)	=	1491.10
Log likelihood = -912.90163	Prob > chi2	=	0.0000

Choice	Coefficient	Std. Error	z	P	95% CI LL	95% CI UL
Longer	-2.7857	0.4972	-5.60	0.000	-3.7602	-1.8113
4xW	-5.1708	0.5649	-9.15	0.000	-6.2781	-4.0636
Quality of life	0.3181	0.2141	1.49	0.137	-0.1015	0.7377
Fluid Restriction	0.6162	0.1898	3.25	0.001	0.2442	0.9882
Hospitalisation	0.1011	0.1481	0.68	0.495	-0.1892	0.3913
Access complications	-1.9107	0.3235	-5.91	0.000	-2.5447	-1.2766
+1 year survival	0.9795	0.2523	3.88	0.000	0.4851	1.4740
+2 year survival	3.2435	0.3181	10.19	0.000	2.6199	3.8670
+4 year survival	4.0736	0.4300	9.47	0.000	3.2307	4.9164

Table S3: Fully interacted model result

Mixed logit model	Number of obs	=	6,588
	LR chi2(20)	=	1690.37
Log likelihood = -1106.5489	Prob > chi2	=	0.0000

Choice	Coefficient	Std. Error	z	P	95% CI LL	95% CI UL
4xW Under 50	-4.2400	0.6758	-6.27	0.000	-5.5646	-2.9154
4xW Over 80	-9.5918	1.0333	-9.28	0.000	-11.6171	-7.5665
4xW 50-80	-8.6178	0.8003	-10.77	0.000	-10.1865	-7.0492
Longer	-3.6377	0.5397	-6.74	0.000	-4.6956	-2.5799
Longer Over 80	-4.2249	0.9954	-4.24	0.000	-6.1758	-2.2739
+1 year survival	0.7827	0.2036	3.85	0.000	0.3837	1.1816
+2 year survival	2.5524	0.2268	11.25	0.000	2.1078	2.9969
+4 year survival	3.2182	0.2766	11.63	0.000	2.6760	3.7603
Longer <30 mins travel	3.3624	0.6307	5.33	0.000	2.1262	4.5985
4xW <30 mins travel	2.9340	0.6546	4.48	0.000	1.6509	4.2171
Fluid Restriction	0.6678	0.1421	4.70	0.000	0.3892	0.9463
Longer 2-5 years on HD	-3.2930	0.4425	-7.44	0.000	-4.1603	-2.4257
4xW & Heart Failure	-0.9857	0.6671	-1.48	0.140	-2.2932	0.3218
Longer & Heart Failure	-4.0402	0.7261	-5.56	0.000	-5.4634	-2.6170
4xW Mild Fatigue	-4.8730	1.0237	-4.76	0.000	-6.8794	-2.8666
Longer Severe Fatigue	-1.3673	0.4405	-3.10	0.002	-2.2307	-0.5038
Longer – Does own obs	2.1598	0.5041	4.28	0.000	1.1718	3.1478
4xW – Does own obs	0.1615	0.5509	0.29	0.769	-0.9183	1.2413
Quality of Life	0.3949	0.1307	3.02	0.003	0.1387	0.6512
Access complications	-1.1941	0.1791	-6.67	0.000	-1.5452	-0.8431
